# Transanal down-to-up dissection of the distal rectum as a viable approach to achieve total mesorectal excision in laparoscopic sphincter-preserving surgery for rectal cancer near the anus: a study of short- and long-term outcomes of 123 consecutive patients from a single Japanese institution

**DOI:** 10.1186/s12957-022-02826-5

**Published:** 2022-11-15

**Authors:** Satoru Kagami, Kimihiko Funahashi, Takamaru Koda, Toshimitsu Ushigome, Tomoaki Kaneko, Takayuki Suzuki, Yasuyuki Miura, Yasuo Nagashima, Kimihiko Yoshida, Akiharu Kurihara

**Affiliations:** Department of General and Gastroenterological Surgery, Toho University Omori Medical Center, 6-11-1 Omorinishi Ota-ku, Tokyo, 143-8541 Japan

**Keywords:** Rectal cancer near the anus, Laparoscopic sphincter preserving surgery, Transanal down-to-up dissection of down-to-up dissection of the distal rectum, Transanal total mesorectal excision, Transanal rectal dissection

## Abstract

**Background:**

In rectal cancer (RC) surgery, the complexity of total mesorectal excision (TME) in laparoscopic sphincter-preserving surgery (lap-SPS) for RC near the anus has been a critical issue. Recently, technical assistance via the anus for complete TME has been receiving attention. This study aimed at clarifying the transanal down-to-up dissection viability for achieving TME in lap-SPS for RC near the anus.

**Methods:**

We evaluated surgical and oncological outcomes of a total of 123 consecutive patients undergoing either a transanal rectal dissection (TARD) under direct vision mobilizing the most difficult portion of TME via the anus or the transanal TME by using an endoscopic system (TaTME) for achieving TME in lap-SPS for RC near the anus between January 2006 and February 2021.

**Results:**

A total of 123 consecutive patients (83 men) with a median age of 66 years (range 33–86 years) were included. TARD and TaTME were performed for 50 (40.7%) and for 73 (59.3%) patients, respectively. Preoperative treatment was performed for 40 (32.5%) patients, resulting in a complete pathological response in 5 (12.5%) patients. Intersphincteric resection was performed significantly more in the TARD group (*p*<0.001). Although the TaTME group needed a longer operative time at the transanal portion (*p*<0.001), the median blood loss was lower (*p*<0.001). Postoperative complications with the Clavien–Dindo classification grade ≧2 developed in 52 (42.3%) patients. Urinary dysfunction and stoma-related complications were found most frequently. More patients needing medication for urinary dysfunction were found in the TARD group, but a significant difference was not observed (10.0% vs. 6.8%, *p*=0.526). The quality of TME was good for almost all patients. Recurrence developed in 18 (14.6%) patients. The 5-year overall survival (OS) and relapse-free survival (RFS) rates in 123 patients were 95.8% and 88.8%, respectively. The 5-year OS and RFS between the two groups were comparable.

**Conclusions:**

Our data suggested that a transanal down-to-up dissection of the distal rectum might be a viable approach in lap-SPS for RC near the anus. Further studies are needed to examine the differences between TARD and TaTME.

## Background

Total mesorectal excision (TME) is the standard procedure for reducing local recurrence after rectal cancer (RC) surgery. Although laparoscopic surgery is beneficial in RC surgery, the difficulty of achieving TME in laparoscopic sphincter-preserving surgery (lap-SPS), especially for RC patients with a narrow pelvis, is a critical issue. Improving visualization at the bottom of the pelvis is very useful for achieving complete TME in lap-SPS in RC patients with a narrow pelvis. In addition, improving visualization at the bottom of the pelvis could lead to short-term clinical advantages, including a lower conversion rate to open surgery and less anastomotic leakage [1–5] when compared with conventional abdominal approaches. Technical assistance via the anus for complete TME in lap-SPS is available. It includes transanal abdominal transanal proctosigmoidectomy with a hand-sewn coloanal anastomosis (TATA). and transanal TME (TaTME) mobilization of the rectum under endoscopy [6]. Since January 2006, we have performed lap-SPS combined with the transanal rectal dissection (TARD) for RC near the anus, wherein the most difficult portion of TME is mobilized down-to-up (“under direct vision”) via the anus [7–9]. Since January 2014, we have performed TaTME, wherein the most difficult portion of TME is mobilized down-to-up via the anus, using an endoscopic system instead of direct vision. This study aimed at evaluating the surgical and oncological outcomes of transanal down-to-up rectal dissection, including TARD and TaTME, when lap-SPS was performed for RC near the anus.

## Methods

### Patients

This study was approved by the Ethics Committee of the Toho University Omori Medical Center (clearance number: M 21325). We performed lap-SPS combined with TARD or TaTME to achieve TME in 137 patients with rectal tumors near the anus between January 2006 and February 2021. In this study, 14 patients have been excluded: five patients who required rectal amputation, two patients with gastrointestinal stromal tumors, two patients with neuroendocrine tumors, four patients with rectal cancer located at >5 cm from the anal verge, and one patient with missing data. Thus, we retrospectively evaluated surgical and oncological outcomes in 123 patients.

### Outcomes

As for surgical outcomes, we evaluated total operation time, blood loss, the number of dissected lymph nodes, the time to catheter removal, and postoperative complications. Postoperative complications were reported according to the Clavien–Dindo classification. Anastomotic leakage was determined by evaluating a combination of symptoms, imaging, and/or radiological findings. The degree of urinary dysfunction was determined by the incidence of re-indwelling urinary catheters, requiring medication and/or clean intermittent catheterization after surgery. As for oncological outcomes, we evaluated the specimen’s pathological quality, including the mesorectum, circumferential resection margin (CRM), distal resection margin from the rectal stump (DM), the incidence of recurrence, overall survival, and relapse-free survival. Gastrointestinal pathologists assessed the specimens. The quality of the mesorectum was defined according to the method by Quirke et al. [10]. The patients were followed up after surgery: blood tests were performed every 3 months, including carcinoembryonic antigen (CEA) and carbohydrate antigen (CA 19-9). Computed tomography (CT) and/or abdominal ultrasonography were performed every 3 months in the first 3 years and every 6 months thereafter to check for cancer recurrence. Local recurrence was defined as any recurrence diagnosed or suspected in the pelvis.

### Indication of transanal down-to-up rectal dissection combined with lap-SPS for RC near the anus

We chose transanal down-to-up rectal dissection to perform lap-SPS for RC near the anus. Intersphincteric resection (ISR) was indicated for lesions <5 cm from the anal verge, excluding clinical stage IV cancer cases. According to the UICC TNM classification of malignant tumors, we first evaluated clinical TNM staging using enhanced CT and magnetic resonance imaging (MRI). For locally advanced tumors with clinical stage N2-3 and/or suspected direct invasion to adjacent organs (including the prostate, vagina, and levator ani muscle), we administered chemoradiation therapy (CRT) with S-1 or neoadjuvant chemotherapy (NAC) before surgery. Subsequently, we evaluated the final TNM staging using enhanced CT and MRI. Patients with obvious findings of direct invasion to adjacent organs were excluded from the indication of lap-SPS with transanal mobilization and underwent rectal amputation instead.

### Surgical procedure

**Fig. 1:**
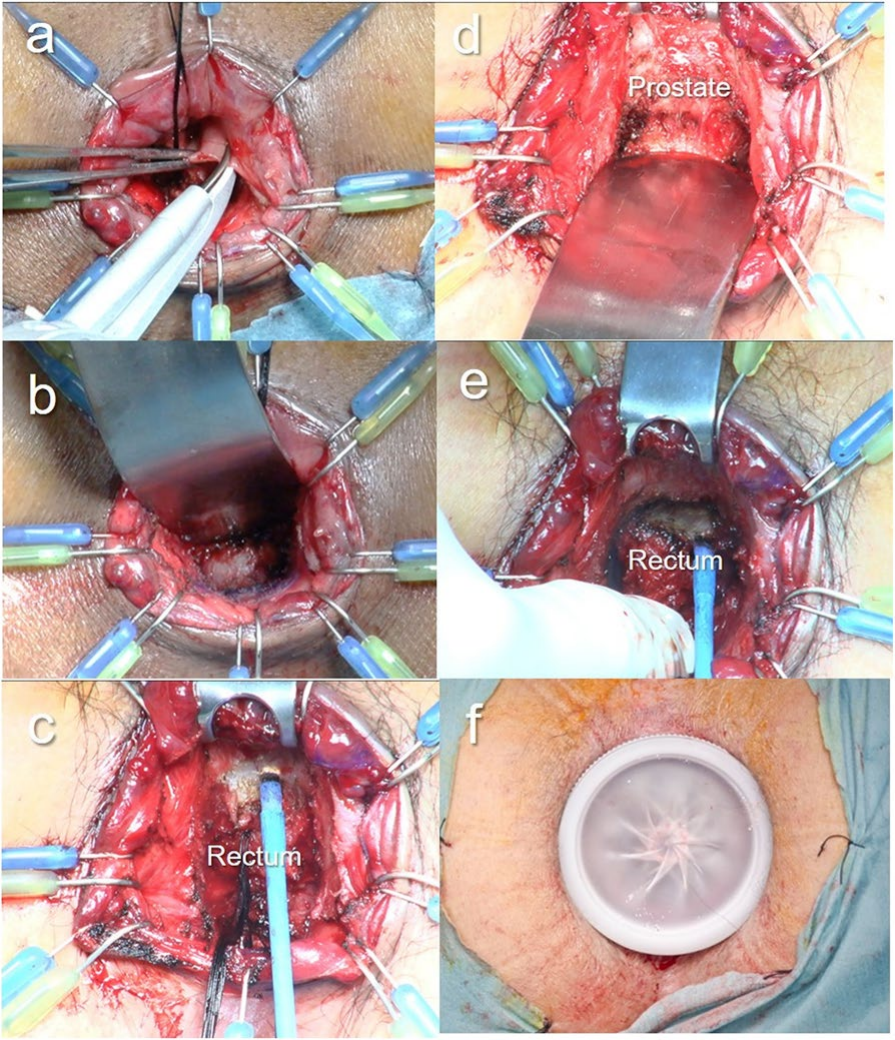
Transanal rectal dissection (TARD) procedure. The distal side at the lower margin of the tumor is closed with a purse-string suture under direct vision, followed by irrigation of the anal canal with 5% povidone-iodine. The division of the rectum is then initiated at the posterior side ≧2 cm distal to the distal margin. First, a circular incision of the rectum is performed by closing the cut end of the rectum with an interrupted suture (**a**). Second, the distal rectum is mobilized proximally while developing a surgical field using a self-holding retractor (Lone Star Retractor) and a spatula. At the posterior side of the rectum, the distal rectum can be easily mobilized using an electronic scalpel and a pusher after incising the ligament between the rectum and the coccyx (**b**). At the anterior side of the rectum, the recto-urethral muscle is incised while developing a surgical field by using a spatula, and then both anterolateral sides of the rectum are dissected. However, where the neurovascular bundle is located, the anterolateral sides of the rectum would be dissected transabdominally (**c** and **d**). Division and mobilization of the rectum, including the mesorectum, is performed as possible until the peritoneal reflection at the anterior side and the rectosacral ligament at the posterior side are identified (**e**). Finally, a lap disc mini is adapted to the anal canal to maintain pressure during laparoscopy (**f**)

The surgical technique for TARD has been previously described [7–9]. First, division of the rectum was initiated at the posterior side: ≥ 2 cm for clinical T2 and 3 cancers or ≥ 1 cm for clinical T1 cancer, distal to the tumor margin [11, 12]. As for the goal of TARD, division and mobilization of the distal rectum (including the mesorectum) are performed “under direct vision,” both anteriorly (until the peritoneal reflection is identified) and posteriorly (until the sacral promontory beyond the rectosacral ligament is nearly reached), using an electronic scalpel and a pusher. However, where the neurovascular bundle was located, we transabdominally dissected both anterolateral sides of the rectum. We immediately converted to rectal amputation if we suspected any findings of direct invasion of the external anal sphincter or levator ani muscles during dissection of the internal and external anal sphincter muscles (Fig. 1).

Contrastingly, for TaTME, division and mobilization of the rectum (including the mesorectum) are performed using an electric scalpel under an endoscope instead of under direct vision. In this series, because all RCs were located ≤ 5 cm from the anal verge, we dissected the distal rectum under direct vision, beyond the anorectal ring, to access the EZ ACCESS ®(Hakko. Co., Ltd. Medical Division, Japan) as a platform for the TaTME.

**Fig. 2:**
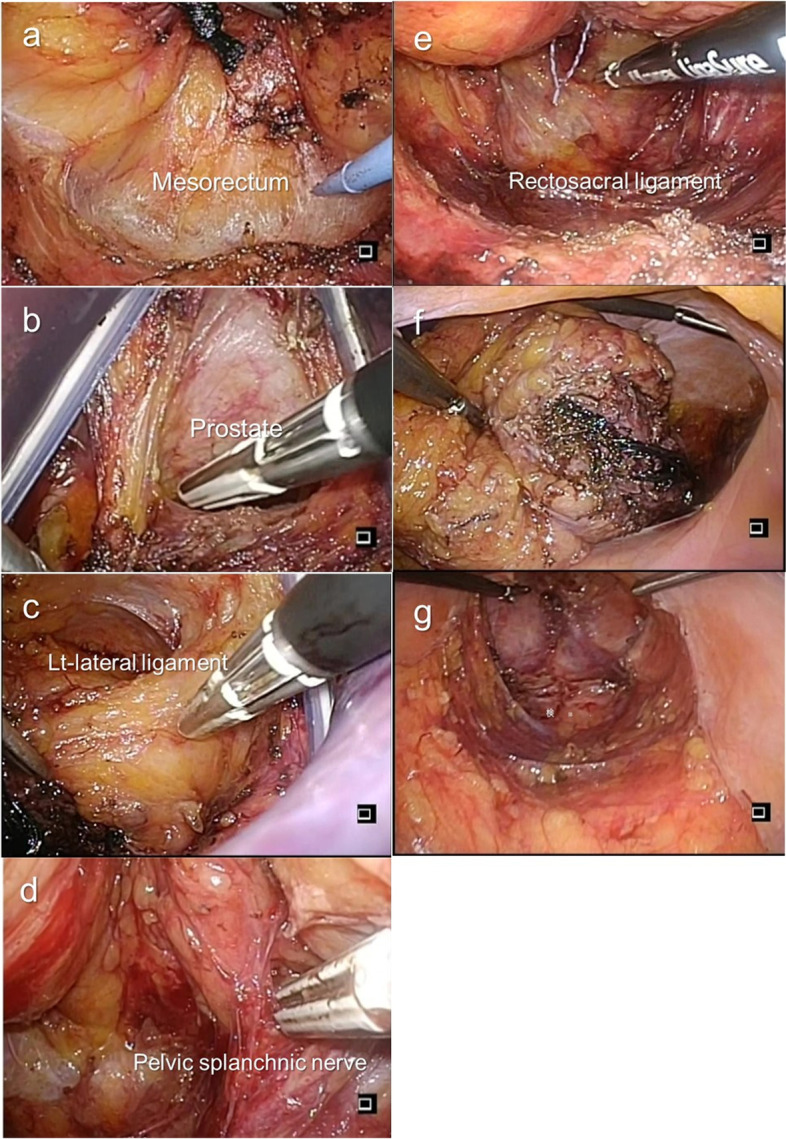
Transanal total mesorectal excision (TaTME) procedure. We usually mobilize the distal rectum at least above the anorectal ring in order to set up the EZ ACCESS platform at the anal canal. We try to dissect until the peritoneal reflection at the anterior side of the rectum is identified, because we often experience difficulty to dissect the anterior side of the rectum through the EZ ACCESS platform

Second, we initiated the laparoscopic procedure. The sigmoid colon mesentery was mobilized, preserving the superior hypogastric plexus. The lymph nodes around the inferior mesenteric artery were dissected with a harmonic scalpel. The inferior mesenteric artery was ligated at a high level using an endoclip. The sigmoid and descending colons were mobilized completely from the sub-retroperitoneal fascia to ensure that the subsequent coloanal anastomosis was free of tension. The splenic flexure was mobilized routinely for coloanal anastomosis. During peritoneal reflection, exposing the seminal vesicles and prostate gland or the posterior of the vagina is relatively easy. On the posterior of the rectum, we mobilized the lower rectum with the mesorectum from the sacrum towards gauze, which was placed at the separated plane between the visceral and parietal endopelvic fascia, via the anus, as a landmark. The lateral ligaments of the rectum are gradually divided with an electric and/or harmonic scalpel from the inner limit of the inferior hypogastric nerve fibers. The rectum, including the total mesorectum, could thus be removed from the pelvic floor. Finally, the colon and rectum were removed from the umbilical wound and resected. Coloanal anastomosis was transanally performed by hand suturing. A diverting ileostoma was created for each procedure. All the procedures were performed by a single team (Fig. 2).

### Statistical analysis

Comparisons between the two groups were performed using chi-square (*χ*^2^) or Fisher’s exact tests for categorical variables and Mann–Whitney *U* tests for continuous variables. Tests of significance were two-sided, and p values <0.05 were considered statistically significant. All data were entered into a computer database and analyzed using EZR version 1.55 [13], which is for R. EZR is a modified version of the R commander designed to add statistical functions frequently used in biostatistics.

## Results

### Patient characteristics

**Fig. 3:**
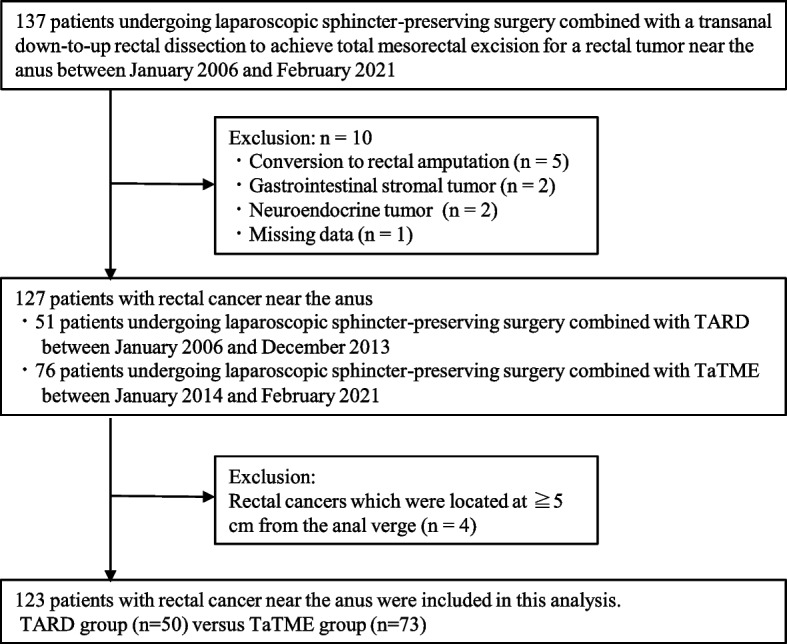
Patient selection of the study population. TARD, transanal rectal dissection; TaTME, transanal total mesorectal excision

The study population consisted of 50 patients who underwent lap-SPS combined with TARD mobilizing the distal rectum (under direct vision) via the anus between January 2006 and December 2013 (TARD group) and 73 patients who underwent lap-SPS combined with TaTME (using an endoscopic system instead of direct vision) via the anus between January 2014 and February 2021 (TaTME group), respectively (Fig. 3). The characteristics of 123 patients are presented in Table 1. The study population consisted of 83 men, with a median age of 66. Preoperative treatments were administered to 40 (32.5 %) of the 123 patients, including 29 patients who received CRT and 11 who received NAC. In respect of NAC, according to preoperatively evaluation of TNM staging by an enhanced CT and MRI, we used to administer NAC for bulky tumors occupying the pelvis and for locally advanced tumors with clinical lymph node metastases and/or suspected of direct invasion to the adjacent organs including the prostate, the vagina, and the levator ani muscle. In this study, we administered NAC for 11 patients in the TaTME group. Specifically, 7 patients had been diagnosed preoperatively with clinical N2 stage, and 3 patients had tumors that were located at the anterolateral side of the rectum and were suspected of direct invasion to the prostate or vagina. In the last patient, the tumor was bulky and had occupied the pelvis although it was categorized as a clinical N1 stage. Five (12.5%) of the 40 patients showed a complete pathological response. TARD and TaTME were performed in 50 (40.7%) and 73 (59.3%) patients, respectively. Among the 50 patients with TARD and 73 patients with TaTME, significantly more men were in the TaTME group (*p* = 0.024). In addition, all 16 patients who received CRT as preoperative treatment were included in the TARD group. No significant differences in age, body mass index, preoperative treatments, TNM factors, or pathological stage (pStage) were observed between the groups. Ultra-low anterior resection and coloanal anastomosis by hand suturing and ISR were performed in 75 (61.0%) and 48 (39.0%) patients, respectively. ISR was performed more frequently in the TARD group than in the TaTME group (58.0% versus 26.0%; *p* <0.001).

**Table 1:** Patients’ characteristics

	Total (*N*=123)	TARD (*n* = 50)	TaTME (*n* = 73)	*P* value
Sex, *n* (%)				0.024
Male	83 (67.5)	28 (56.0)	55 (75.3)	
Female	40 (32.5)	22 (44.0)	18 (24.7)	
Age^a^	66 (33–86)	63.5 (33–77)	66 (41–86)	0.298
Body mass index^a^	21.9 (16.7–41.8)	21.5 (16.7–37.2)	22.5 (16.8–41.8)	0.376
Preoperative treatment, *n* (%)				1.000
Positive	40 (32.5)	16 (32.0)	24 (32.9)	
CRT	29 (23.6)	16 (32.0)	13 (17.8)	
NAC	11 (8.9)	0 (0.0)	11 (15.1)	
Negative	83 (67.5)	34 (68.0)	49 (67.1)	
Resection of the AIS				
ISR	48 (39.0)	29 (58.0)	19 (26.0)	<0.001
Ultra-LAR with CAA	75 (61.0)	21 (42.0)	54 (74.0)	
Tumor related factor				
T, *n* (%)				0.299
1b	21 (17.0)	10 (20.0)	11 (15.1)	
2	38 (30.9)	14 (28.0)	24 (32.9)	
3	54 (43.9)	23 (46.0)	31 (42.5)	
4a	5 (4.1)	0 (0.0)	5 (6.8)	
pCR	5 (4.1)	3 (6.0)	2 (2.7)	
N, *n* (%)				1.000
Positive	33 (26.8)	13 (26.0)	20 (27.4)	
Negative	90 (73.2)	37 (74.0)	53 (72.6)	
M, *n* (%)				1.000
Positive	2 (1.6)	1 (2.0)	1 (1.4)	
Negative	121 (98.4)	49 (98.0)	72 (98.6)	
pStage, *n* (%)				0.874
I	52 (42.3)	22 (44.0)	30 (41.1)	
II	33 (26.8)	12 (24.0)	21 (28.8)	
III	31 (25.2)	12 (24.0)	19 (26.0)	
IV	2 (1.6.)	1 (2.0)	1 (1.4)	
pCR	5 (4.1)	3 (6.0)	2 (2.7)	

*CRT* chemoradiation therapy, *NAC* neoadjuvant chemotherapy, *AIS* the anal internal sphincter muscle, *CAA* coloanal anastomosis, *pCR* pathological complete response, *TARD* transanal rectal dissection, *TaTME* transanal total mesorectal excision

^a^ Median (range)

### Surgical outcomes

Table 2 shows the surgical outcomes of the patients. The median operative time of the perineal portion was significantly longer in the TaTME group than in the TARD group (95 versus 145 min; *p*<0.001), but the total operation time was not significantly different between the groups. Median blood loss was significantly lower in the TaTME group than in the TARD group (277 versus 85.0 ml; *p*<0.001), and the median number of dissected lymph nodes between the groups was comparable (9 nodes versus 10 nodes; *p*=0.295). The median period for urinary catheter removal following surgery was 5 days (range 3–16 days) in the TaTME group, which was significantly longer than the 4 days (range 2–18 days) in the TARD group (*p*<0.001). Postoperative complications (Clavien–Dindo classification grade ≥ 2) occurred in 52 (42.3%) of the 123 patients: 18 (36.0%) patients in the TARD group and 34 (46.6%) patients in the TaTME group (*p*=0.269). In addition, severe postoperative complications (Clavien–Dindo classification grade ≥ 3) occurred in 24 (19.5%) of the 123 patients: 10 (20.0%) patients in the TARD group and 14 (19.2%) patients in the TaTME group (*p*=0.910).

**Table 2:** Surgical outcomes

	Total (*N*=123)	TARD (*n*=50)	TaTME (*n*=73)	*P*-value
Total operative time, minute^a^	431 (291–906)	417 (291–625)	441 (317–906)	0.098
Perineal portion	122 (26–261)	95 (26–174)	145(62–261)	< 0.001
Abdominal portion	316 (179–589)	319 (214–504)	302 (179–589)	0.336
Blood loss, ml^a^	110 (0–1097)	277 (0–1097)	85 (0–765)	< 0.001
No. of dissected LN, *n*^a^	9 (0–38)	9 (2–38)	10 (0–33)	0.295
Period to catheter removal^a^, day	5 (2–18)	4 (2–18)	5 (3–16)	< 0.001
Postoperative complication (C-D ≧grade 2), *n* (%)				0.269
Positive	52 (42.3)	18 (36.0)	34 (46.6)	
Negative	71 (57.7)	32 (64.0)	39 (53.4)	
Postoperative severe complication (C-D ≧grade 3), *n* (%)				0.910
Positive	24 (19.5)	10 (20.0)	14 (19.2)	
Negative	99 (80.5)	40 (80.0)	59 (80.8)	

*LN* lymph node, *C-D* the Clavien-Dindo classification, *TARD* transanal rectal dissection, *TaTME* transanal total mesorectal excision

^a^ Median (range)

No cases of mortality occurred in our cohort. Urinary dysfunction and stoma-related complications were the most frequent complications, occurring in 16 (13.0%) patients., and leakage occurring in 11 (8.9%) patients (including one patient in the TARD group requiring reoperation because of major anastomotic leakage). As for urinary dysfunction, 13 (10.6%) patients required urinary catheter re-indwelling, including one (0.8%) patient who required clean intermittent catheterization. Finally, 10 patients (8.1%) required medication for urinary dysfunction. Outlet obstruction and high-output stoma, as stoma-related complications, developed frequently. No significant differences in postoperative complications were observed between the groups (Table 3).

**Table 3:** Postoperative complication of the Clavien-Dindo classification grade ≧2

	Total (*N*=123)	TARD (*n*=50)	TaTME (*n*=73)	*P*-value
Leakage, *n* (%)	11 (8.9)	2 (4.0)	9 (12.3)	0.197
Pelvic abscess, *n* (%)	8 (6.5)	3 (6.0)	5 (6.8)	1.000
Stoma-related complication, *n* (%)	16 (13.0)	6 (12.0)	10 (13.7)	1.000
Outlet obstruction	7 (5.7)	2 (4.0)	5 (6.8)	
High output	8 (6.5)	3 (6.0)	5 (6.8)	
Parastomal abscess	2 (1.6)	1 (2.0)	1 (1.4)	
Collapse	1 (0.8)	1 (2.0)	0 (0)	
Gangrenous pyoderma	1 (0.8)	0 (0)	1 (1.4)	
Ileus, *n* (%)	9 (7.3)	6 (12.0)	3 (4.1)	0.157
Urinary dysfunction	16 (13.0)	5 (10.0)	11 (15.1)	0.587
Urinary catheter re-indwelling, *n* (%)	13 (10.6)	5 (10.0)	8 (11.0)	1.000
Medication, *n* (%)	10 (8.1)	5 (10.0)	5 (6.8)	0.526
CIC, *n* (%)	1 (0.8)	1 (2.0)	0 (0)	1.000
Others, *n* (%)	3 (2.4)	1 (2.0)	2 (2.7)	1.000

*CIC* clean intermittent catheterization, *TARD* transanal rectal dissection, *TaTME* transanal total mesorectal excision

### Oncological outcomes

We achieved complete or nearly complete TME in all patients in this study. In addition, we identified a negative CRM in all the patients. As for DM, the median distance of the distal resection margin from a tumor was longer in the TARD group than in the TaTME group (20.0 versus 15.0 mm; *p*=0.112). Five (4.1%) patients had a complete pathological response to the preoperative treatment. Finally, excluding the five patients with complete pathological responses, the number of patients with pStages I, II, and III was 52 (42.3%), 33 (26.8%), and 31 (25.2%), respectively. Finally, two (1.6%) patients were diagnosed with pStage IV (Table 4).

**Table 4:** Oncological outcomes

	Total (*N*=123)	TARD *(n*=50)	TaTME (*n*=73)	*P*-value
TME				0.896
Complete	119 (96.7)	48 (96.0)	71 (97.3)	
Nearly complete	4 (3.3)	2 (4.0)	2 (2.7)	
Incomplete	0 (0)	0 (0)	0 (0)	
CRM				
Positive, *n* (%)	0 (0)	0 (0)	0 (0)	
Negative, *n* (%)	123 (100)	50 (100)	73 (100)	
DM				1.00
Positive, *n* (%)	1 (0.8)	0 (0)	1 (1.4)	
Negative	122 (99.2)	50 (100)	72 (98.6)	
Distance^a^ mm (range)	15 (0.5–45)	20 (3–45)	15 (0.5–45)	0.112
Final stage, *n* (%)				0.874
I	52 (42.3)	22 (44.0)	30 (41.1)	
II	33 (26.8)	12 (24.0)	21 (28.8)	
III	31 (25.2)	12 (24.0)	19 (26.0)	
IV	2 (1.6)	1 (2.0)	1 (1.4)	
pCR	5 (4.1)	3 (6.0)	2 (2.7)	

*CRM* circumferential resection margin, *DM* distal resection margin, *pCR* pathological complete response, *TARD* transanal rectal dissection, *TaTME* transanal total mesorectal excision

^a^ Median

**Fig. 4:**
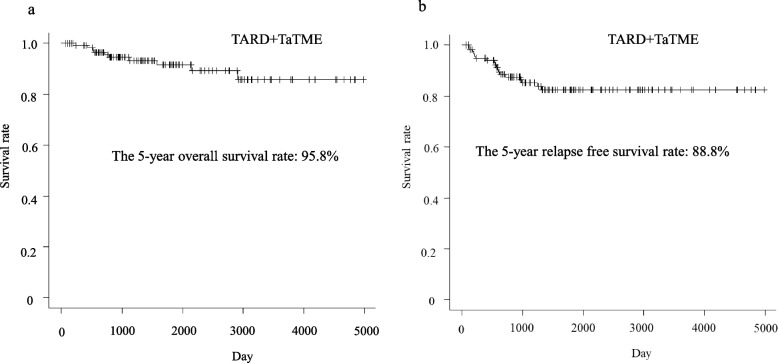
Survival curves of 123 patients undergoing a transanal down-to-up dissection of the distal rectum in laparoscopic sphincter-preserving surgery for rectal cancer near the anus. TARD, transanal rectal dissection; TaTME, transanal total mesorectal excision

**Fig. 5:**
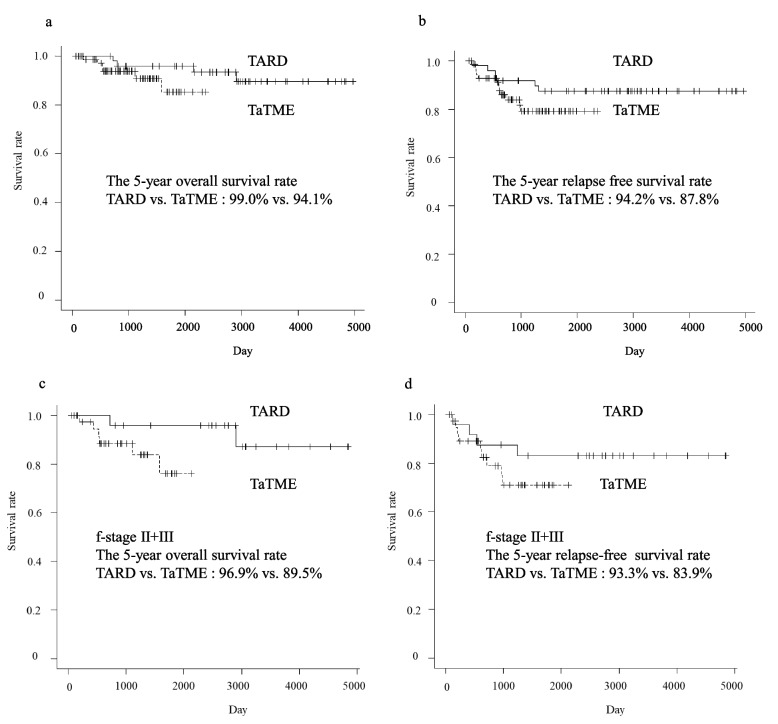
Comparison of survival curves between the transanal rectal dissection (TARD) group and the transanal total mesorectal excision (TaTME) group

Recurrence developed in 18 (14.9%), with a median observation time of 1440 (range 69–4975 days) days: one patient with a complete pathological response, four patients with pStage I, three patients with pStage II, and ten patients with pStage III. Recurrence rates in pStages I, II, and III were 7.7% (4/52 patients), 9.1% (3/33 patients), and 32.3% (10/31 patients), respectively, and a high recurrence rate was found in locally advanced cancers, such as pStage III cancers. Lung metastases (4.9%) developed most frequently, followed by pelvic lymph nodes (4.1%). The median period until recurrence was 18.5 (range 4–41) months. No significant differences in each of the recurrence patterns and the median period until recurrence were observed between the groups (Tables 5 and 6). Overall survival (OS) and relapse-free survival (RFS) are shown in Fig. 4. The 5-year OS and RFS rates in the 123 patients who underwent transanal down-to-up dissection of the distal rectum were 95.8% and 88.8%, respectively. The 5-year OS rates between the two groups were comparable (99.0% in the TARD group versus 94.1% in the TaTME group; *p*=0.151). The 5-year RFS rates between the two groups were comparable (94.2% in the TARD group versus 87.8% in the TARD group; *p*=0.219). As for the patients with final Stages II and III, the 5-year OS and RFS rates in the TaTME group were lower than that in the TARD group, respectively. Considering both 5-year OS and RFS rates, there were no differences between the groups (Fig. 5).

**Table 5:** Recurrence rate of each pathological stage

Recurrence, *n* (%)		Total	TARD	TaTME
pStage	I	4/52 (7.7)	2/22 (9.1)	2/30 (6.7)
	II	3/33 (9.1)	1/12 (8.3)	2/21 (9.5)
	III	10/31 (32.3)	3/12 (25.0)	7/19 (36.8)
pCR		1/5 (20.0)	0/3 (0)	1/2 (50.0)
Total		18/121^a^ (14.9)	6/49 (12.2)	12/72 (16.7)

*pCR* pathological complete response, *pStage* pathological stage, *TARD* transanal rectal dissection, *TaTME* transanal total mesorectal excision

^a^ Excluding two patients who were diagnosed with pathological Stage IV finally

**Table 6:** Recurrence pattern

	Total (*N*=123)	TARD (*n*=50)	TaTME (*n*=73)	*P*-value
Recurrence, *n* (%)				0.607
Negative	105 (85.4)	44 (88.0)	61 (83.6)	
Positive	18 (14.6)	6 (12.0)	12 (16.4)	
Lung	6 (4.9)	2 (4.0)	4 (5.5)	
Liver	1 (0.8)	0 (0)	1 (1.4)	
Liver+ local	1 (0.8)	0 (0)	1 (1.4)	
Lung + Liver	1 (0.8)	1 (2.0)	0 (0)	
Bone	1 (0.8)	0 (0)	1 (1.4)	
Para-aorta lymph node	1 (0.8)	0 (0)	1 (1.4)	
Lymph nodes in the pelvis	5 (4.1)	2 (4.0)	3 (4.1)	
Local	1 (0.8)	1 (2.0)	0 (0)	
Wound	1 (0.8)	0 (0)	1 (1.4)	
The period until recurrence^a^, month (range)	18.5 (4–41)	17.5 (4-41)	19 (4–32)	0.778
Observation time^a^, day (range)	1440 (69–4975)	2900 (670–4975)	1051 (69–2361)	<0.001

*TARD* transanal rectal dissection, *TaTME* transanal total mesorectal excision

^a^ Median

## Discussion

Achieving complete TME in lap-SPS for mid- and low-RC is a significant oncological issue. Therefore, various strategies to achieve complete TME have been developed. Marks et al. [14] first reported favorable outcomes of cancer of the distal 3 cm of the true rectum by TATA with a hand-sewn coloanal anastomosis. Marks et al. [15, 16] then reported the usefulness of TATA in lap-SPS for rectal cancer. Kanso et al. [17] evaluated the short- and long-term outcomes of 138 patients treated with the primary perineal approach, compared with the primary abdominal approach, in ISR for low RC. The researchers concluded that a primary perineal approach could be considered the standard strategy for lap-SPS for low RC because of reduced operative time and similar short- and long-term outcomes. In addition, a randomized trial by Denost et al. [18] suggested that perineal rectal dissection was a new standard in lap-SPS for low RC since perineal rectal dissection reduced the risk of positive CRM in low RC.

In 2006, we started TARD combined with lap-SPS as a strategy to achieve complete TME in lap-SPS for RC near the anus—a procedure that mobilizes the most different portion of TME, “under direct vision,” in a down-to-up manner via the anus [7, 8]. We have been performing TaTME, using an endoscopic system, in lap-SPS for RC near the anus since January 2014. Recently, TaTME have been extensively studied worldwide. In this study, we aimed to evaluate the feasibility of transanal down-to-up dissection of the distal rectum, including TARD, to assist in achieving complete TME in lap-SPS for RC near the anus, assessing the surgical and oncological outcomes of these two procedures.

Regarding surgical outcomes, TaTME significantly reduced blood loss when compared with TARD. In contrast, using TARD to dissect the distal rectum under direct vision might be more straightforward, because the operative time of the perineal portion in TARD was shorter than that of TaTME; however, the shorter operative time of the perineal portion did not affect the total operative times of either procedure, in this study.

Second, in this study, no cases of mortality occurred; however, we experienced relatively high rates of postoperative complications, with 42.3% of patients displaying Clavien–Dindo classification grade ≥ 2 and 19.5% of patients displaying Clavien–Dindo classification grade ≥ 3. Urinary dysfunction and stoma-related complications were the most common postoperative complications, followed by anastomotic leakage. According to previous studies, the incidence of postoperative complications in the TARD group is nearly equivalent. Kanso et al. [17] reported overall morbidity and severe morbidities in ISR with a primary perineal approach in 47% and 16% of patients, respectively. Also, Denost et al. [18] reported that surgical morbidity occurred in 12% of lap-SPS cases.

In a study by Roodbeen et al. [19], postoperative complications (Clavien–Dindo classification grade ≥ 2 and ≥ 3) developed in 13 (31.7%) and 9 (22.0%) patients (out of 41 patients) with TaTME, respectively. In another study by Munini et al. [20], the frequency of Clavien–Dindo classification grade ≥ 2 was 28.9% in TaTME patients, while grade ≥ 3 classifications were absent. In contrast, Hallam et al. [21] reported more favorable outcomes: postoperative complications (Clavien–Dindo classification grades ≥ 2 and ≥ 3) occurred in 14% (10/70 patients) and 11% (8/70 patients) of patients, respectively. In a recent systematic review and meta-analysis by An et al. [22], the overall morbidity of TaTME was 30%, including leakage at 6.8%.

Colorectal surgeons are aware that urinary morbidity is an important clinical issue during down-to-up dissection of the rectum via the anus. In other studies, the rate of urinary injury has been reported to be 1–11%. Klein et al. [23] reported that, although the urinary function was preserved in 89% of 115 patients, a urethral injury occurred in one patient, while six (5%) patients required permanent urinary catheterization. In addition, Sylla et al. [24] analyzed urologic injuries in TaTME patients and reported 34 urethral, two ureteral, and three bladder injuries occurring during TaTME, performed over 7 years by 32 surgical teams.

Although none of our patients incurred urethral injury in this study, urinary dysfunction developed in 16 (13.0%) patients, including 10 (8.1%) patients who required medication for urinary dysfunction. In general, a transanal down-to-up approach to the rectum, including TARD and TaTME, is likely to injure the urethra and its associated neurovascular bundle. In particular, anterior dissection of the distal rectum is, technically, very difficult because of anatomical complexity. Attention must be paid to the neurovascular bundle located at the anterolateral side of the rectum to avoid urinary morbidity after surgery. In TARD, we performed the dissection transabdominally rather than transanally, for the anterolateral side of the rectum, where the neurovascular bundle was located. In contrast, taking advantage of good vision under endoscopy in TaTME, we could easily perform transanal dissection around the neurovascular bundle. For these reasons, urinary morbidity might have occurred more frequently in the TaTME group than in the TARD group. In addition, 10.0% (5 patients) of patients in the TARD group required medication for urinary dysfunction. This was thought to be due to blind dissection at the rectum’s anterior side under direct vision.

Leakage is a critical issue in transanal down-to-up rectal dissection procedures. Previous studies have reported an anastomotic leakage rate of between 5.5 and 17.9% [19–23, 25].

The international TaTME registry reports a morbidity rate of 35.4%, including an anastomotic failure rate of 15.7%, a pelvic abscess rate of 4.7%, an anastomotic fistula rate of 0.8%, a chronic sinus rate of 0.9%, and an anastomotic stricture rate of 3.6%. The registry also reported that male sex, obesity, smoking, diabetes mellitus, tumors >25 mm, excessive intraoperative blood loss, manual anastomosis, and prolonged perineal operative time were independent risk factors for anastomotic failure [26].

In this study, anastomotic leakage occurred in 8.9% of patients, including one who required reoperation because of ischemia at the anastomotic site [27]. However, for other patients with leakage, we could only treat the patients conservatively, with interventional radiological drainage. This might be because diverting ileostomas were created in all patients. In addition, in this study, stoma-related complications occurred more frequently than expected, where most patients had outlet obstruction and/or a high-output stoma. Stoma-related complications could be associated with the fact that we created a stoma at the ileum and not at the transverse colon for all patients.

Third, focusing on oncological outcomes is also important to clarify the feasibility of down-to-up dissection for RC. In particular, local recurrence is of great concern. We achieved complete or nearly complete TME in all patients. In addition, we obtained safe surgical margins, including the CRM and DMs in both groups. Jiang et al. conducted a systematic review and meta-analysis [28], showing that TaTME had more positive CRM and DM advantages than laparoscopic TME. We believe that transanal down-to-up dissections have some benefits. First, we could convert the procedure to rectal amputation whenever any direct invasion of the adjacent organs (such as the vagina, prostate, and seminal vesicles) was suspected during mobilization of the distal rectum [29]. Second, with down-to-up dissections under direct vision, we could obtain an adequate distant margin from the end of the tumor. For patients who needed ISR to preserve the anus for RC near the anus, we obtained an adequate distance from the end of the RC. For five patients in whom the tumor was suspected of invading the external sphincter muscle or the adjacent organs (such as the prostate and vagina), we could choose an adequate surgery for rectal amputation from an oncological perspective.

In this study, recurrence developed in 14.6% (18/123) of patients. Although no significant difference in recurrent organs was found between the two groups, we observed a high recurrence rate of 32.3% even in pStage III, compared with 7.7% in pStage I and 9.1% in pStage II. Metastasis to the lungs and local recurrence, including the pelvis lymph nodes, was frequent. Wasmuth et al. [30] reported a high local recurrence rate of 7.6% (12/157 patients) at a median follow-up of 20 months, including eight unexpected patterns of multifocal or extensive local recurrences, resulting in the abandonment of TaTME in Norway. van Oostendorp et al. [31] reported the short-term outcomes of 120 patients with TaTME performed at 12 centers. The overall local recurrence rate was 10%, with a mean interval to recurrence of 15.2 months. Multifocal local recurrence was present in eight of 12 patients. In a prolonged cohort (266 patients), overall multifocal local recurrence rate was 5–6%. The researchers stated that multifocal local recurrence may be closely associated with suboptimal TaTME execution. In both studies, most local recurrences were multifocal or extensive. Our study did not encounter the same degree of multifocal or extensive local recurrence reported in the literature.

In a prospective multicenter study in Denmark [2], the dissection plane was mesorectal in 60% of the cases, intra-mesorectal in 28%, and muscular in 12%. Non-micro-radicality was observed in 8% of cases, although microscopic and macroscopic residual tumors were observed in 6% and 2%, respectively. Although the local recurrence rate was 3.5% (4/115 patients), one patient had multifocal recurrence at a median follow-up of 23 months. A systematic meta-analysis review by Jiang et al. [28], examining pathological outcomes of transanal versus laparoscopic TME for RC, reported that TaTME had better outcomes for positive CRM than laparoscopic TME. However, Caycedo-Marulanda et al. [32] reported that a positive CRM increased the risk of local recurrence 4.2 times more than a negative CRM. Local recurrence is likely closely associated with positive CRM. Therefore, we emphasize cognizance regarding the development of multifocal or extensive local recurrence in a transanal down-to-up dissection of the rectum, which could be closely associated with a positive CRM. Preoperative treatment is important for patients with locally advanced RC.

Finally, this study found a favorable survival rate, with a 5-year OS rate of 95.8% and a 5-year RFS rate of 88.8%. In comparing the two groups, the 5-year OS and RFS were comparable. 

Marks et al. [16] reported a 5-year OS rate of 90% (regarding long-term outcomes) after a transanal approach to TME for RC. In addition, Denost et al. [33] reported that the 5-year OS and DFS rates of transanal low rectal dissection were 87.0% and 72%, respectively. Recently, we found studies evaluating the long-term outcomes of TaTME. Hol et al. [34] reported that the 5-year DFS and OS rates in 159 consecutive patients with TaTME were 81% and 77.3%, respectively. In addition, Ourô et al. [35] reported that the 5-year DFS and OS rates in 44 patients with TaTME were 87.0% and 81%, respectively.

Recent meta-analyses [36–41] suggest that transanal down-to-up dissection procedures, including TARD and TaTME, might be acceptable in lap-SPS for mid-and low-RC. However, we currently reason that oncological safety in a transanal down-to-up dissection for RC is still controversial. We must wait for the results of the COLOR III [42] and ETAP-GRECCAR 11 TRIAL [43] studies to further evaluate the efficacy and safety of TaTME.

This study had some limitations. First, this study was conducted at a single institution in Japan, and the data were retrospectively analyzed using a small sample size. As a transanal down-to-up dissection of the distal rectum to achieve complete TME is considered a difficult technique even for colorectal surgeons, the quality of the techniques could improve with increasing surgical experience. These experiences might affect surgical and oncological outcomes. However, we could not evaluate the outcomes of 123 patients, especially for the TARD group, in which only 50 patients could be evaluated in this study. The small sample size of the TARD group may cause some bias in surgical and oncological outcomes. Second, this study used a one-team approach for all patients. If two teams had performed the surgery, we might have reduced both operative time and blood loss because of improved visualization of the anterior side of the distal rectum. Third, we were not able to evaluate the accurate distance between the anal verge and the tumor by reviewing the medical and operative records. Finally, we should have evaluated functional outcomes following surgery to assess further the efficacy and safety of the procedures we performed.

## Conclusions

Our data suggest that transanal down-to-up rectal dissections, including TARD, might be an acceptable procedure for achieving TME in lap-SPS for RC near the anus from both a surgical and an oncological perspective. However, to further confirm the possible superiority of TaTME for advanced RC, large-scale multicenter randomized controlled trials are warranted.

## Data Availability

The data used during the current study are available from the corresponding author on reasonable request.

## Abbreviations

CA 19-9: Carbohydrate antigen
CEA: Carcinoembryonic antigen
CRT: Chemoradiation therapy
CRM: Circumferential resection margin
CT: Computed tomography
DFS: Disease-free survival
DM: Distal resection margin
ISR: Intersphincteric resection
lap-SPS: Laparoscopic sphincter-preserving surgery
MRI: Magnetic resonance imaging
NAC: Neoadjuvant chemotherapy
OS: Overall survival
RC: Rectal cancer
TARD: Transanal rectal dissection under direct vision
TATA: transanal abdominal transanal proctosigmoidectomy with a hand-sewn coloanal anastomosis
TaTME: Transanal TME
TME: Total mesorectal excision
